# Single-port laparoscopic surgery for parasitic cystic teratoma of the greater omentum and bilateral ovarian teratomas: a case report

**DOI:** 10.1093/jscr/rjad021

**Published:** 2023-01-31

**Authors:** Akihiko Misawa, Eizo Kimura, Aiko Oka, Kiyono Osanai, Atsushi Suzuki

**Affiliations:** Department of Obstetrics and Gynecology, Kosei Hospital, 2-25-1 Wada, Suginami-ku, Tokyo 166-0012, Japan; Department of Obstetrics and Gynecology, Kosei Hospital, 2-25-1 Wada, Suginami-ku, Tokyo 166-0012, Japan; Department of Obstetrics and Gynecology, Kosei Hospital, 2-25-1 Wada, Suginami-ku, Tokyo 166-0012, Japan; Department of Obstetrics and Gynecology, Kosei Hospital, 2-25-1 Wada, Suginami-ku, Tokyo 166-0012, Japan; Department of Obstetrics and Gynecology, Kosei Hospital, 2-25-1 Wada, Suginami-ku, Tokyo 166-0012, Japan

## Abstract

Teratomas of extragonadal origin are extremely rare. The most common extragonadal site is the greater omentum. A 36-year-old woman was referred to our department for treatment of bilateral ovarian tumors. A 3- to 4-cm mass was observed in each ovary; bilateral ovarian mature cystic teratoma was diagnosed through imaging studies. The patient underwent single-port laparoscopic surgery, during which we found not only bilateral ovarian mature cystic teratomas but also an omental tumor macroscopically similar to the ovarian teratomas. The ovarian and omental teratomas were successfully removed through single-port laparoscopic surgery. The histopathological diagnosis of the omental tumor was parasitic mature cystic teratoma.

## INTRODUCTION

Mature cystic teratoma is a common type of ovarian tumor, accounting for 27–44% of all ovarian tumors and 35–58% of benign ovarian cysts [1, 2]. However, extragonadal teratomas are extremely rare, occurring most commonly in the greater omentum. Teratomas of the greater omentum are usually asymptomatic, and most are discovered unexpectedly during abdominal surgery for other indications [3].

Single-port surgery is thought to reduce postoperative pain and the need for analgesics and produce better cosmetic results [4]. Single-port laparoscopic surgery for ovarian cysts is more effective than three-port laparoscopic surgery [5].

We present the case of a woman who underwent single-port laparoscopic surgery for bilateral ovarian mature cystic teratomas with an omental tumor discovered incidentally during surgery.

## CASE REPORT

The patient was 36 years old, gravida 2 para 1, had a regular menstrual cycle and no dysmenorrhea and was not on low-dose estrogen and progestin. At age 23, she had laparoscopic surgery for bilateral ovarian cysts. At 36, she visited another hospital for persistent abdominal and back pain; thus, bilateral ovarian cysts were diagnosed, and she was referred to our hospital for further examination.

**Figure 1:**
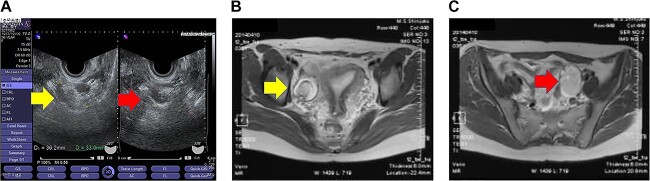
Imaging studies of the pelvis. (**A**) Transvaginal ultrasound image showing a right ovarian cyst (left arrow) and a left ovarian cyst (right arrow). (**B**) Axial T2-weighted MRI showing a right ovarian cyst (arrow). (**C**) Axial T2-weighted MRI showing a left ovarian cyst (arrow).

Transvaginal ultrasonography revealed a 3–4-cm diameter mass on each ovary (Fig. 1A) and a normal-sized uterus. Magnetic resonance imaging (MRI) revealed bilateral ovarian cystic tumors with fat tissue (Fig. 1B and C). The laboratory values, including levels of tumor markers (e.g. CA-125, CA 19-9 and alpha-fetoprotein), were within normal limits. The patient was diagnosed with mature cystic teratomas of the bilateral ovaries. Because the ovarian tumors were relatively small during initial examination, a 3-month follow-up visit was recommended. During the follow-up visit, the patient requested surgery for persistent lumbago. Single-port laparoscopic surgery, which is indicated for benign ovarian cysts, was recommended based on surgical indications at our department.

**Figure 2:**
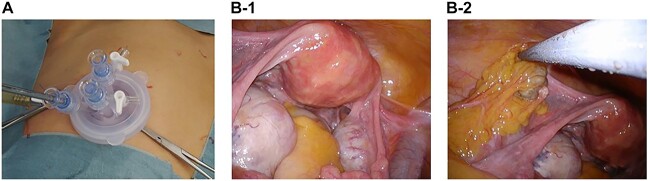
Laparoscopic views and pathological findings. (**A**) Single-port system for laparoscopic viewing: Lap Disc Mini® (Hakko Corporation, Osaka, Japan) and EZ Access® (Hakko Corporation). (**B**-1) The uterus, which is normal-sized, and the ovaries, which are swollen because of the presence of teratomas. (**B**-2) Omental tumor, resembling a teratoma.

The patient was placed in a steep Trendelenburg position for surgery after general anesthesia was administered. To facilitate the surgical procedures, a uterine manipulator was inserted. An approximately 3-cm abdominal incision was made in the umbilicus, and the skin was retracted using a Lap Disc Mini® (Hakko Corporation, Osaka, Japan) and EZ Access® (Hakko Corporation). A 5-mm flexible laparoscope was used to maintain pelvic visualization. To use the conventional laparoscopic atraumatic grasping forceps, two 5-mm trocars were inserted through the Lap Disc Mini® (Fig. 2A). Bilateral ovarian mature cystic teratomas and an omental tumor that was macroscopically similar to the ovarian teratomas were discovered intraoperatively (Fig. 2B-1 and B-2). No other masses were found in the abdominal cavity. We performed single-port laparoscopic surgery to remove the bilateral ovarian cysts and the omental tumor. The patient was discharged from the hospital on the fifth postoperative day after an uneventful postoperative course. One year after surgery, follow-up showed that the patient was in good health.

**Figure 3:**
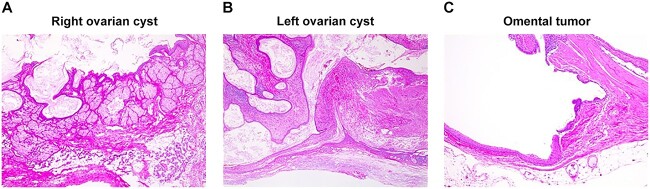
Pathological findings: right ovarian cyst (**A**), left ovarian cyst (**B**) and tumor in the greater omentum (**C**). (A, B) Ectodermal derivatives including the central nervous tissue and mesodermal derivatives including cartilage and adipose tissue in the ovarian tumor. (C) Ectodermal derivatives including squamous epithelium, respiratory epithelium and goblet cells were observed in the omental tumor.

The ovarian tumor was a unilocular cystic tumor with sebaceous material on the left side, sebaceous material mixed with hair on the right side and sebaceous material interspersed with solid nodules and cysts. A unilocular cystic tumor containing teeth, sebaceous material and squamous epithelium comprised the omental tumor. Histopathologically, the ovarian tumor contained ectodermal derivatives such as central nervous tissue and mesodermal derivatives such as cartilage and adipose tissue. The omental tumor contained ectodermal derivatives such as squamous epithelium, respiratory epithelium and goblet cells (Fig. 3). The omental tumor was a mature cystic teratoma, and no ovarian interstitial tissue was found within the cystic structure. The clinical diagnosis revealed parasitic dermoid cyst.

## DISCUSSION

Mature cystic teratomas (benign cystic teratomas or dermoid cysts) are believed to develop from germ cells in the mature gonads. However, teratomas of the greater omentum are extremely rare and their case is unknown [6]. Omental teratomas are more common in women than in men, usually during their reproductive years, but some appear during childhood and at 60–70 years of age in women. Three major theories about the origin of omental teratomas have been proposed: (1) that primary teratomas of the omentum originate from displaced germ cells, (2) they develop from supernumerary ovary tissue of the omentum and (3) autoamputation of ovarian dermoid cysts leads to secondary implantation in the greater omentum [6]. Another hypothesis is that the tumor completely separates from its pedicle and transforms into a parasitic teratoma. Another hypothesis is that ovarian cystic teratomas rupture into the abdominal cavity, causing peritonitis and adhesions, and small fragments of ruptured teratomas become implanted in the omentum. Secondary implantation tumors are the most commonly found in the greater omentum [6]. The absence of ovarian tissue is a criterion for diagnosing secondary omental implants [7].

There was no ovarian tissue in our patient’s omental tumor. She had undergone laparoscopic bilateral ovarian cystectomy 13 years earlier (details of the previous surgery are unknown); thus, we hypothesize that the omental teratoma was a parasitic teratoma caused by the implantation of cyst contents ruptured during the previous surgery.

At our institution, single-port laparoscopic surgery is used for ovarian tumors, for ectopic pregnancies and diagnostic laparoscopy. The procedure is minimally invasive, with only one 3-cm-long wound at the navel. Because it is difficult to place two forceps or devices and a laparoscope in a single port, proficiency in single-port surgery is gained gradually. To avoid interference between forceps and devices, we find it easier to operate two forceps or devices by crossing them in the abdominal cavity rather than using them in parallel. With this, we have successfully resected majority of bilateral ovarian cysts. It allowed us to observe the entire omentum in the abdominal cavity with a laparoscope and displace the greater omentum through the 3-cm navel wound and observe it directly in our patient.

To summarize, we present a rare case of mature cystic teratoma of the greater omentum, which is thought to be a parasitic teratoma caused by previous surgery implantation of ruptured cyst contents. The omental teratoma was found incidentally during laparoscopic surgery for bilateral ovarian cyst. Single-port laparoscopic surgery successfully removed the bilateral ovarian cysts and the omental tumor.

## Data Availability

All data generated or analysed during this study are included in this published article.
